# Change in Daily Negative Affect Across 20 Years

**DOI:** 10.1093/geroni/igab046.825

**Published:** 2021-12-17

**Authors:** Susan Charles, Jennifer Piazza, Jonathan Rush

**Affiliations:** 1 University of California, Irvine, Irvine, California, United States; 2 California State University, Fullerton, California State University, Fullerton, California, United States; 3 University of Victoria, Victoria, British Columbia, Canada

## Abstract

The current study examined levels of daily NA among people (N=413) who participated in three waves of the National Study of Daily Experiences (~1996; ~2008; ~2017). At each wave, participants reported how often they had experienced six negative emotional experiences every day for eight consecutive days. Cross-sectional analyses at each time-point show age-related decreases in NA. Trajectories over time, however, were moderated by age (Est = .006, SE = .002, p = .001), revealing a curvilinear pattern. Among people who were 25-50 years-old at the first wave, daily NA decreased over time, with decreases more pronounced among the younger adults. For people at least 50 years-old at the start of the study, daily NA increased over time, with the slopes steepest for older adults. Findings indicate that cross-sectional and longitudinal age-related patterns in NA differ when examining data collected from 1996 to 2017.

